# The causality between premature ventricular contraction and heart failure

**DOI:** 10.1002/joa3.13218

**Published:** 2025-01-10

**Authors:** Naoya Kataoka, Teruhiko Imamura

**Affiliations:** ^1^ Second Department of Internal Medicine University of Toyama Toyama Japan

To editor:

Ogiso and colleagues demonstrated that nonsustained ventricular tachycardia (NSVT) is associated with an increased risk of heart failure hospitalization in patients without structural heart disease.^1^ However, several critical concerns warrant further discussion.

A comprehensive methodology detailing the approach to confirm the absence of structural heart disease should be provided. Importantly, various cardiac pathologies with preserved left ventricular ejection fraction cannot be definitively excluded without comprehensive testing. For instance, epicardial cardiomyopathy cannot be ruled out without advanced diagnostic modalities, such as cardiac magnetic resonance imaging and genetic testing.^2^

The burden of premature ventricular contractions (PVCs) is a well‐documented contributor to systolic dysfunction, with a commonly proposed threshold exceeding 20%.^3^ In this study, however, the total number of PVCs was categorized into tertiles,^1^ which may limit the precision of the analysis.

Differentiating PVCs with aberrant conduction in patients with atrial fibrillation using Holter electrocardiography presents significant challenges.^4^ A detailed description of the methodology used to distinguish these phenomena is essential for reproducibility and validity. Additionally, the rationale for administering class III antiarrhythmic agents in patients reportedly free of structural heart disease remains unclear and requires elucidation.

The causal relationship between NSVT and the development of heart failure remains ambiguous.^1^ Notably, most heart failure hospitalizations occurred within 1 year of observation. It is plausible that patients experiencing elevated left ventricular end‐diastolic pressure may develop NSVT as a secondary manifestation. In such cases, subclinical heart failure could potentially be identified through detailed investigations, including chest X‐rays, B‐type natriuretic peptide levels, and comprehensive echocardiography.

Finally, if PVCs serve merely as bystanders of underlying cardiac pathology, the efficacy of aggressive therapeutic interventions targeting NSVT and PVCs in improving clinical outcomes becomes questionable.

## CONFLICT OF INTEREST STATEMENT

The authors declare no conflicts of interest.

## Data Availability

The manuscript does not include any original data.
